# In vitro evaluation of PLGA loaded hesperidin on colorectal cancer cell lines: an insight into nano delivery system

**DOI:** 10.1186/s12896-024-00882-1

**Published:** 2024-08-02

**Authors:** Narges Yaghoubi, Amir Gholamzad, Tahere Naji, Mehrdad Gholamzad

**Affiliations:** 1 Faculty of Pharmacy and Pharmaceutical Sciences, Tehran Medical Sciences, Islamic Azad University, Tehran, Iran; 2 Farhikhtegan Medical Convergence Sciences Research Center, Farhikhtegan Hospital Tehran Medical Sciences, Islamic Azad University, Tehran, Iran; 3 Department of Basic Sciences, Faculty of Pharmacy and Pharmaceutical Sciences, Tehran Medical Sciences, Islamic Azad University, Tehran, Iran; 4 Department of Microbiology and Immunology, Faculty of Medicine, Tehran Medical Sciences, Islamic Azad University, Tehran, Iran

**Keywords:** PLGA, Hesperidin, Nano particles, Drug delivery, Colorectal cancer

## Abstract

**Background:**

Colorectal cancer is a common disease worldwide with non-specific symptoms such as blood in the stool, bowel movements, weight loss and fatigue. Chemotherapy drugs can cause side effects such as nausea, vomiting and a weakened immune system. The use of antioxidants such as hesperidin could reduce the side effects, but its low bioavailability is a major problem. In this research, we aimed to explore the drug delivery and efficiency of this antioxidant on the HCT116 colorectal cancer cell line by loading hesperidin into PLGA nanoparticles.

**Materials and methods:**

Hesperidin loaded PLGA nanoparticles were produced by single emulsion evaporation method. The physicochemical properties of the synthesized hesperidin-loaded nanoparticles were determined using SEM, AFM, FT-IR, DLS and UV-Vis. Subsequently, the effect of the PLGA loaded hesperidin nanoparticles on the HCT116 cell line after 48 h was investigated by MTT assay at three different concentrations of the nanoparticles.

**Result:**

The study showed that 90% of hesperidin were loaded in PLGA nanoparticles by UV-Vis spectrophotometry and FT-IR spectrum. The nanoparticles were found to be spherical and uniform with a hydrodynamic diameter of 76.2 nm in water. The release rate of the drug was about 93% after 144 h. The lowest percentage of cell viability of cancer cells was observed at a concentration of 10 µg/ml of PLGA nanoparticles loaded with hesperidin.

**Conclusion:**

The results indicate that PLGA nanoparticles loaded with hesperidin effectively reduce the survival rate of HCT116 colorectal cancer cells. However, further studies are needed to determine the appropriate therapeutic dosage and to conduct animal and clinical studies.

## Introduction

Colorectal cancer occurred in all or the parts of the large intestine, located in the lower end of the digestive system, and includes various types of carcinoma tumors, gastrointestinal stromal tumors, lymphomas, and sarcomas. Initial cases manifest as non-cancerous polyps, including various adenomas, inflammatory polyps, hyperplastic polyps, sessile serrated polyps, and common serrated adenomas, often without any symptoms. However, they can be detected through screening, which is why healthcare professionals recommend screening for individuals over the age of 50 [1]. Non-specific symptoms of this type of cancer may include blood in the stool, changes in bowel movements, weight loss, and fatigue, which require precise differential diagnosis through tissue sampling (biopsy) via sigmoidoscopy or colonoscopy [2].

Significant risk factors for colorectal cancer include age, gender, high intake of fat and sugar, red meat and processed food consumption, alcohol consumption, obesity, and lack of physical activity [2]. Another important factor is a family history of colorectal cancer, where the risk of developing this type of cancer increases two to three times for individuals with a family history of the disease [2]. Overall, worldwide, over 1 million people are diagnosed with colorectal cancer each year. It is the second most common cancer in women (9.2% of diagnoses) and the third most common cancer in men (10% of diagnoses), making it the fourth leading cause of cancer-related deaths after lung, stomach, and liver cancer [3].

The prevalence of colorectal cancer in Iran shows that, on average, 58.5% of men and 1.4% of women are diagnosed with this cancer throughout their lives, regardless of age [4]. The treatment of this type of cancer includes various drugs, surgery, and chemotherapy, all of which come with challenges and side effects that impact the patient’s acceptance and quality of life [3]. Studies have shown that particularly in the first three years after recovery, the physical and mental quality of life for survivors of this disease is lower compared to age-matched individuals without cancer of the same age [4]. Some of these challenges include fatigue, sleep problems, increased risk of infection, sensory neuropathy, sexual dysfunction, incontinence, anxiety and depression, bruising and oral ulcers, delayed wound healing, and bleeding, and high blood pressure [5].

Hesperidin, a flavonoid found in citrus fruits, belongs to the class of flavonoids and has a wide range of applications in preventing cardiovascular diseases [6], neuronal degradation [7], and cancer [8]. It has been shown that the anticancer effects of hesperidin are associated with its antioxidant and anti-inflammatory activities [6, 9]. Hesperidin interacts with various cellular targets and prevents the proliferation of cancer cells through the induction of apoptosis and cell cycle arrest [8]. Additionally, there is substantial evidence suggesting the promising role of this compound in inhibiting tumor metastasis, angiogenesis, and chemo resistance [8].

Hesperidin plays a crucial role in regulating oxidative stress, inflammation, and cancer cell death. It induces apoptosis through NF-kB, mTOR, and PI3K/AKT pathways and regulates mediators and enzymes involved in tumorigenesis. Combining hesperidin with other drugs may enhance cancer treatment [10–12]. However, the clinical use of hesperidin has been limited due to its low solubility in water and body fluids, low bioavailability, and limited absorption, posing challenges in effective drug delivery to the target tissue [13–15]. Hence, nanotechnology is employed for optimal use and targeted delivery of hesperidin. This technology can overcome the challenges of low solubility, bioavailability, and drug absorption, as these parameters significantly impact the treatment of various diseases [16]. Nanoformulations, including Hesperetin-TPGS micelles, Hesperetin-PC compounds (notably, hesperetin is an aglycone form of hesperidin), chitosan nanoparticles containing hesperidin, silver nitrate nanoparticles containing hesperidin, titanium dioxide nanoparticles containing hesperidin, and gold nanoparticles containing hesperidin, have been studied in animal and clinical trials to improve solubility in water, antioxidant activity, and enhance the absorption and bioavailability of this compound [17, 18]. The present study aims to investigate the effect of PLGA nanoparticles containing hesperidin on the HCT116 cell as a cancer cell line.

## Material and method

### Materials

Poly (lactic-co-glycolic acid) (PLGA) and poloxamer 407, dimethyl sulfoxide, and hesperidin were purchased from Sigma and Aldrich. Ascorbic acid, fetal bovine serum, trypsin-EDTA, 3– (4, 5-dimethylthiazal-z-yl)-2, 5-diphenylterazolium (MTT) were purchased from Sigma and Aldrich. Dulbecco’s modified Eagle’s medium (DMEM, high glucose) was purchased and used without further purification from commercial suppliers (Sigma-Aldrich).

### Preparation of hesperidin-PLGA nanoparticles

A solution of native hesperidin and PLGA was prepared by dissolving them in DMSO and sonicated for 20 min. A homogenous solution was created by magnetically agitating at 1000 RPM for 30 min. Poloxamer 407 was added as a stabilizer. The bioactive ingredient and PLGA were added in the dispersion phase. The emulsion was mixed with deionized distilled water, agitated overnight, and centrifuged for 15 min to separate unbound stabilizer and active compounds. The concentration of unbound hesperidin in the aqueous phase was determined using a UV-Vis spectrophotometer at 283 nm.

### Drug encapsulation and drug loading calculations

The quantification of the encapsulated hesperidin on the PLGA nanoparticles was conducted using the following equations:

$$\eqalign{& {\rm{Drug}}\,{\rm{encapsulation}}\,\left( {\rm{\% }} \right) \cr & = \,\left( {{\rm{Initial}}\,{\rm{amount}}\,{\rm{of}}\,{\rm{Hes - supernatant}}\,{\rm{free}}\,{\rm{amount}}\,{\rm{of}}\,{\rm{Hes}}} \right) \cr & /{\rm{Initial}}\,{\rm{amount}}\,{\rm{of}}\,{\rm{Hes}} \times 100 \cr}$$

$$\eqalign{& {\rm{Drug}}\,{\rm{loading}}\,\left( {\rm{\% }} \right) \cr & = \,\left( {{\rm{Initial}}\,{\rm{amount}}\,{\rm{of}}\,{\rm{Hes - supernatant}}\,{\rm{free}}\,{\rm{amount}}\,{\rm{of}}\,{\rm{Hes}}} \right) \cr & /{\rm{Weight}}\,{\rm{of}}\,{\rm{Hes}}\,{\rm{Nanoparticles}} \times 100 \cr}$$

Afterwards, the pellet was then re-suspended in a volume of 20 mL of deionized distilled water in order to conduct further evaluations.

### Nanoparticle analysis 

AFM, SEM, DLS, and FT-IR analyses were performed to verify the efficacy and caliber of the generated nanoparticles.

### Cell culture

The HCT116 cell line was acquired from the Pasteur Institute Cell Line Bank (Tehran, Iran). The cells were cultured in Dulbecco’s Modified Eagle’s Medium (DMEM) supplemented with 10% fetal bovine serum (FBS). all falcons were incubated in a humidified environment at 37 °C, with an atmosphere consisting of 5% CO_2_. The medium was refreshed every 2 days.

### Cell viability assay

The HCT116 cells were cultured in DMEM medium supplemented with antibiotics (penicillin-streptomycin and gentamicin) and 10% FBS. They were incubated in a humidified incubator at a temperature of 37 °C, with an atmosphere containing 5% CO_2_. The cytotoxicity of hesperidin nanoparticles was assessed by MTT assay. The HCT116 cells were cultured in 96-well culture plates at a density of 1 × 10^4^ cells per well and incubated at 37 °C for 24 h. Subsequently, the cells were subjected to treatment with the provided samples. To this aim, the media was then aspirated from each well, and serum-free medium containing serial dilutions of hesperidin, nanoparticles and hesperidin nanoparticles were added in three dosages (1, 5, 10 µg/mL). After incubating for an additional 4 h, the cell media was replaced with 100 µL fresh media and incubated for 48 h. After replacing with 100 µL fresh medium containing MTT (final concentration 0.5 mg/ml), the plate was incubated at 37 ˚C for 4 h. After incubation, the medium was replaced with 100 µL/well DMSO to dissolve the formazan products, followed by measuring the absorbance of each well at 570 nm, then color intensity of the formazan solution using the microplate spectrophotometer, and the relative cell viability was obtained using following equation (mean% ± SD, *n* = 3).

$$\eqalign{{\rm{Relative}}\,{\rm{cell}}\,{\rm{viability}} & \cr & {\rm{ = }}\,{\rm{Ab}}{{\rm{s}}_{{\rm{sample}}}}/{\rm{Ab}}{{\rm{s}}_{{\rm{control}}}}\, \times 100 \cr}$$

### Statistical analysis

The experiments were conducted in triplicate, and the data were presented as the mean value and standard deviation. In order to establish statistical significance, one-way analysis of variance (ANOVA) and t-test were performed using GraphPad Prism software and *p* < 0.05 was considered significant.

## Results

### FT-IR analysis of loaded hesperidin

The peaks at cm^− 1^ 3478, 2944, 1649, 1606, 1520, and 1064 correspond to the stretching bonds of O-H, C-H, C = O, C = C, and C-O, respectively, present in hesperidin. The peak at cm^− 1^ 1760 confirms the presence of PLGA in the nanostructure, which corresponds to the stretching bond of ester C = O.

It should be noted that since the peaks of hesperidin compounds are observable without any changes in the nanoparticles, it confirms that hesperidin has been loaded onto PLGA without any structural changes.

**Fig. 1:**
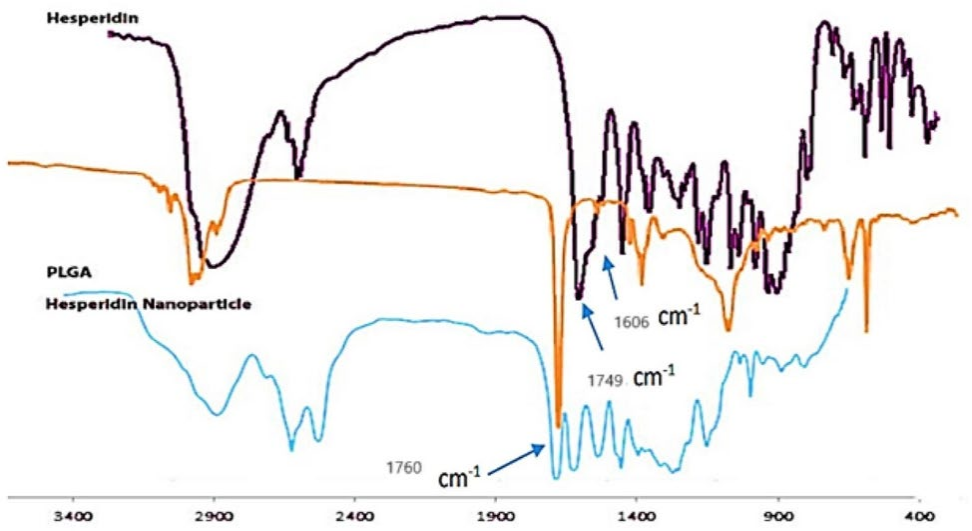
*Comparison of the infrared absorption spectra of hesperidin*,* PLGA polymer*,* and nanoparticles containing hesperidin in terms of cm-1.* The infrared absorption spectrum of nanoparticles containing hesperidin in terms of cm^-1^

The infrared absorption spectrum of hesperidin exhibits characteristic shifts due to the presence of different functional groups. For example, peaks at 916 and 1606 cm-1 can be attributed to the stretching vibrations of O-H, C-H, C = O, C = C, and C-O bonds, respectively. Additionally, in the region of cm-1 1649, the presence of C = O functional groups in the PLGA material is indicated. The absorption spectra of hesperidin and PLGA nanoparticles containing hesperidin are mostly similar, but they have small differences, which could be due to the slight interaction between hesperidin and the PLGA matrix (Figs. 1).

### SEM and AFM

**Fig. 2:**
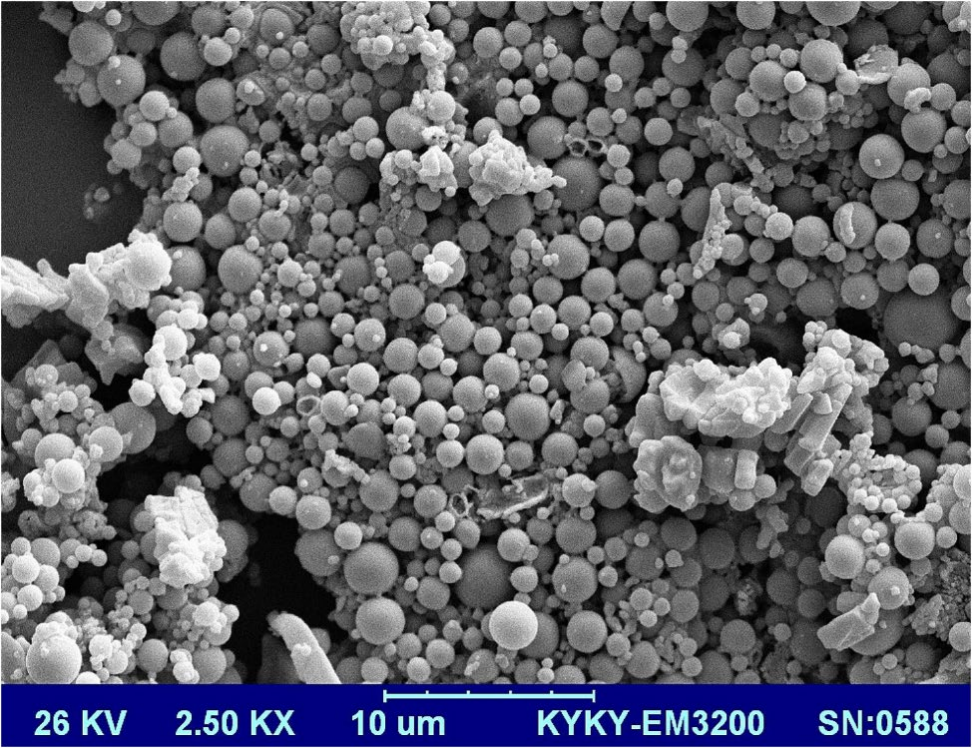
spherical and relatively uniform PLGA Loaded hesperidin

**Fig. 3:**
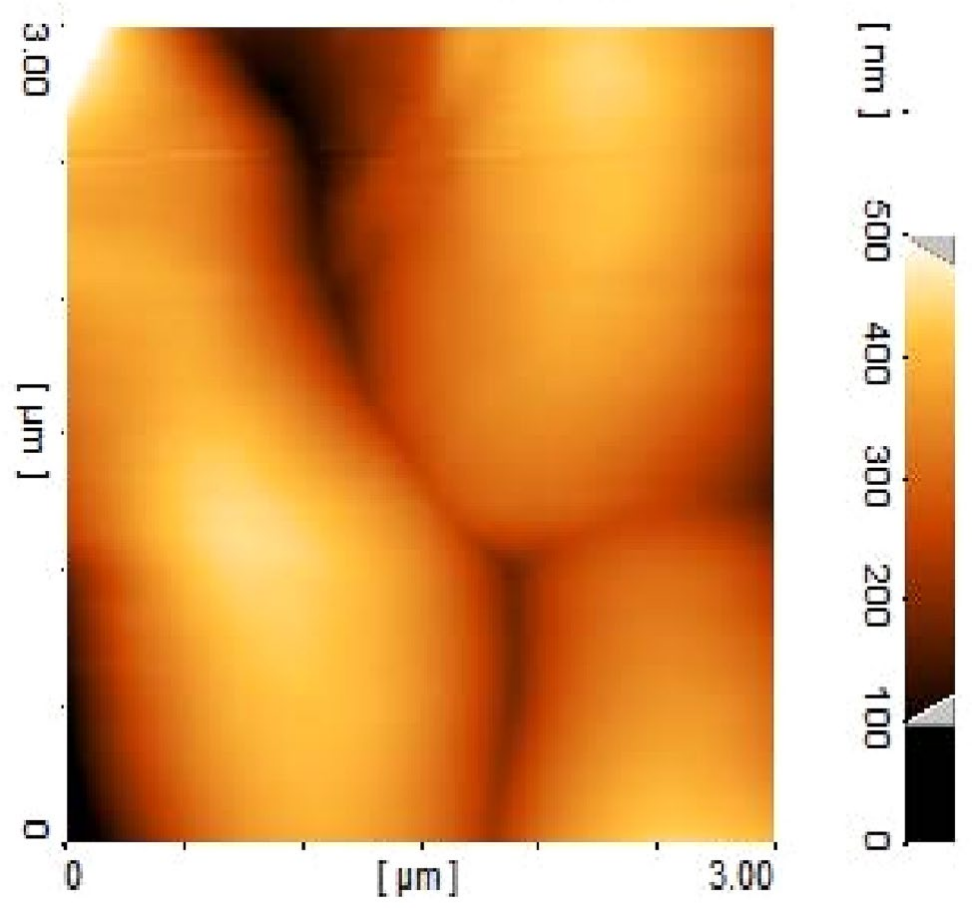
AFM (atomic-force microscopy) image for morphology analysis

The SEM and AFM image of hesperidin loaded nanoparticles in Figs. 2 and 3 shows spherical and relatively uniform nanoparticles with an approximate size of 50 nm.

### Dynamic light scattering (DLS)

**Fig. 4:**
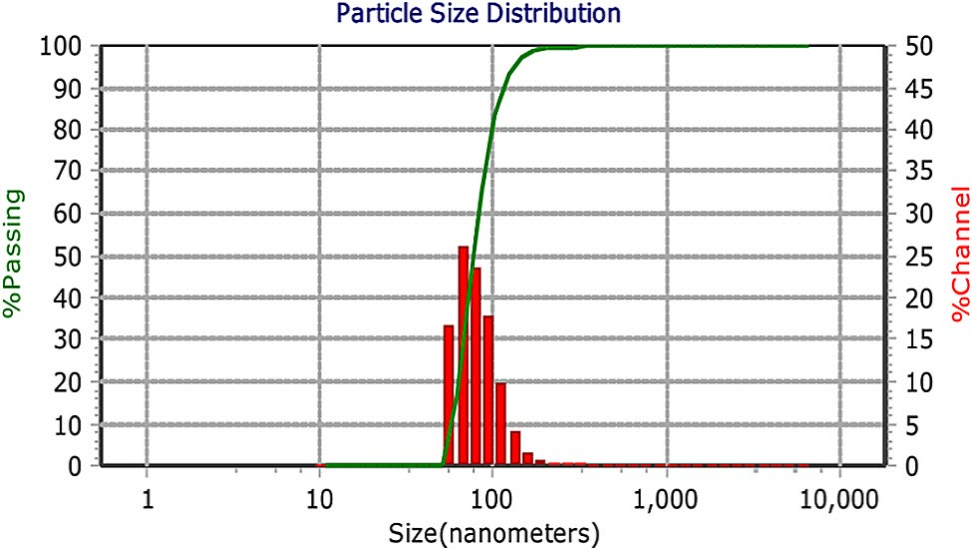
The size distribution of nanoparticles containing Hesperidin in nanometers is characterized by the highest frequency within the range of hydrodynamic diameter of 76.2 nm. PDI = 3.44

**Fig. 5:**
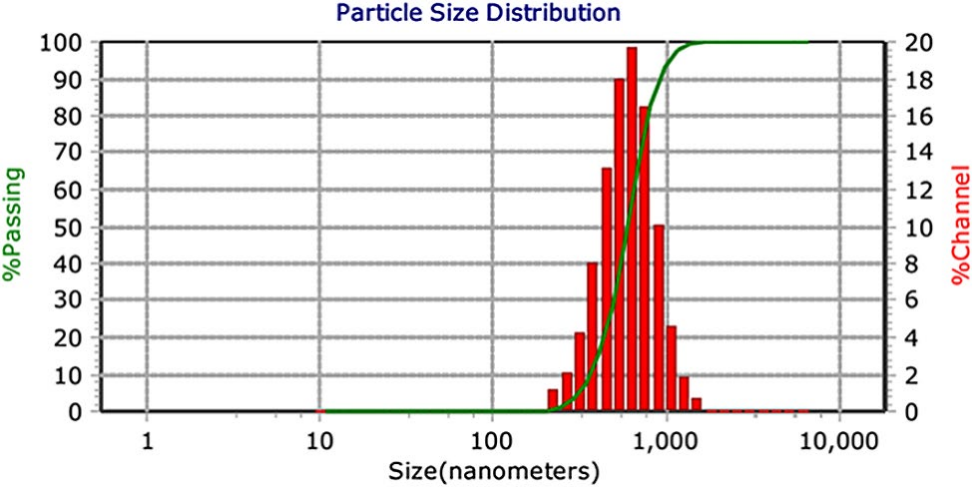
The particle size distribution of Hesperidin material in nanometers is observed to be aggregated, with particles exhibiting the highest frequency within the range of hydrodynamic radius of 595 nm, PDI = 0.976

The DLS results for PLGA nanoparticles are shown in the Figs. 4 and 5. This test was conducted at a temperature of 25 degrees Celsius and at a pH of 7.4. The hydrodynamic diameter of the nanoparticles in aqueous state was estimated to be 76.2 nm. Since Hesperidin is insoluble in water, it forms aggregated particles with a hydrodynamic radius of 595 nm. The particle size values obtained from DLS results are larger than the actual particle size because the device also calculates the water shell radius. However, in electron microscopy analysis, nanoparticles are measured as individual units in a dry state.

### Drug encapsulation and drug loading

**Fig. 6:**
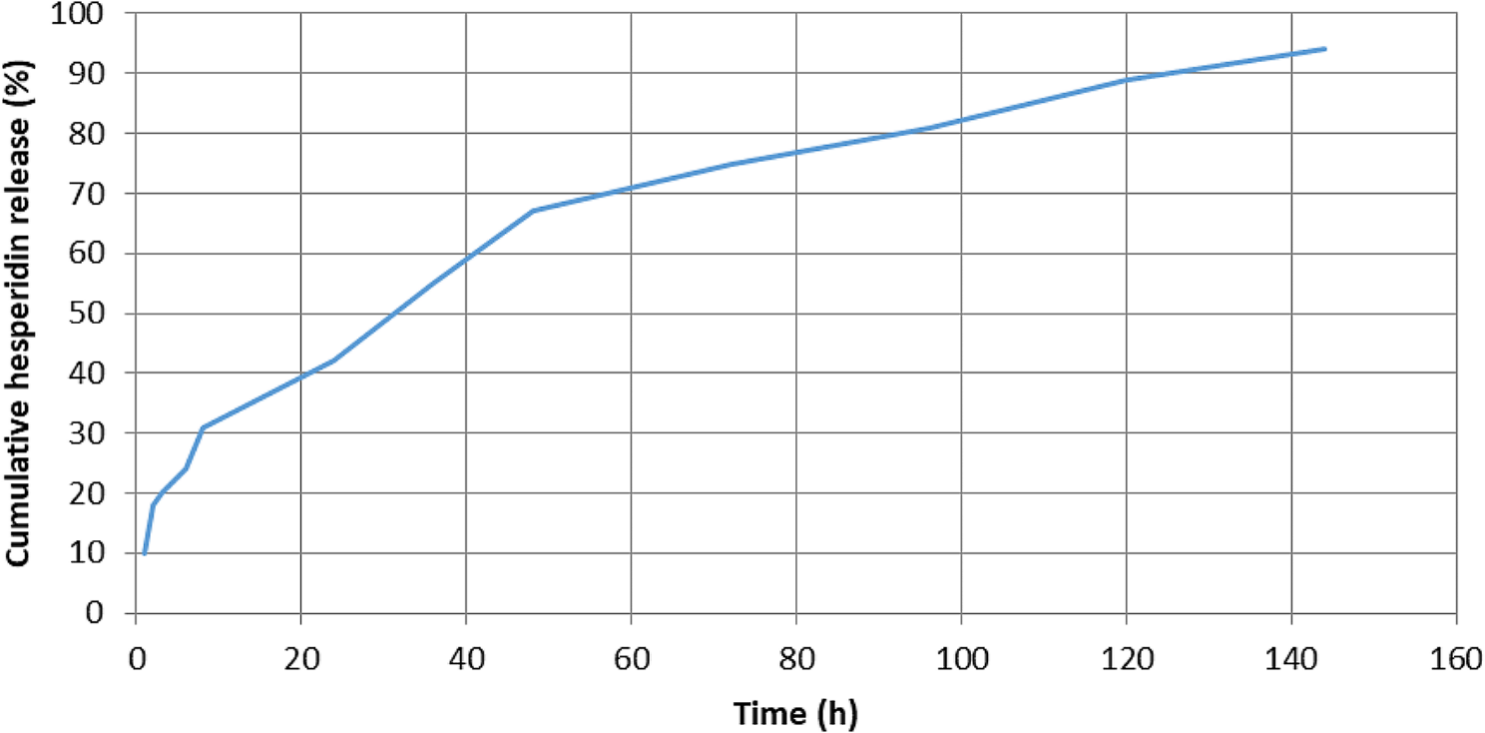
The controlled release percentage of Hesperidin from PLGA nanoparticles over time was studied. In the initial 60 h of release, approximately 70% of Hesperidin was released from the nanoparticles. Subsequently, in the following 84 h, the release rate decreased, with approximately 23% of Hesperidin being released

The drug release rate was evaluated at neutral pH, at a temperature of 37 degrees Celsius, for a duration of 144 h. Additionally, the loading of Hesperidin was confirmed using visible-ultraviolet spectroscopy, despite a peak at a wavelength of 374 nm. In this nanoparticle carrier, the loaded drug amount and encapsulation efficiency were calculated to be 19% and 90%, respectively. Furthermore, the drug release rate under physiological conditions was reported to be approximately 93% after 144 h of administration. The initial hours of drug release showed a slower release rate. What is crucial in controlled drug delivery systems is that the drug is released over a specific period and in a defined and required amount (Fig. 6).

### MTT assay

**Fig. 7:**
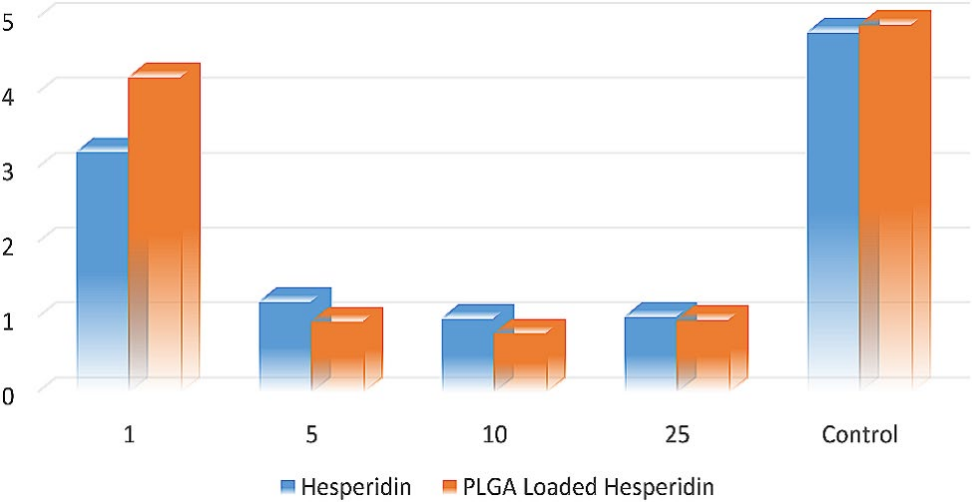
The average percentage of survival of colorectal cancer cells at different concentrations of nanoparticles containing hesperidin compared to control groups (*n* = 3) at 48 h. For a concentration of 10 µg/ml of nanoparticles, there was a significant difference (*p* < 0.001) compared to the control groups

The MTT colorimetric assay is used to investigate the toxicity effects of Hesperidin loaded nanoparticle on the growth and proliferation of cancer cells. PLGA alone does not exhibit any toxicity towards cancer cells. Hesperidin, by itself, does not show cellular penetration have low capability or inhibitory function. However, when hesperidin is encapsulated within the nanoparticle, its ability to effect on cancer cells increased. (Fig. 7)

## Discussion

Colorectal cancer is a common and deadly disease that is influenced by environmental and genetic factors. Most patients are diagnosed with colorectal cancer after the onset of symptoms. Symptoms include rectal bleeding, blood in the stool, changes in bowel habits, constipation, diarrhea, abdominal pain, weight loss and unexplained anemia due to iron deficiency [1–3].

The main treatment methods for this disease include surgery, chemotherapy and radiotherapy. Chemotherapeutic agents used in the treatment of colorectal cancer include capecitabine, a prodrug of fluorouracil and a thymidylate synthase inhibitor; oxaliplatin, an alkylating and cross-linking agent; fluorouracil, an antimetabolite analogue of pyrimidine and a thymidylate synthase inhibitor; and irinotecan, which effectively acts on the S phase of the cell cycle and causes double-stranded DNA breaks. These drugs have significant side effects, including nausea, vomiting, loss of appetite, hand-foot syndrome, bone marrow suppression, and renal and dermatologic complications [4, 5].

Chemotherapy medications used to treat colorectal cancer, such as fluorouracil, raltitrexed, irinotecan, and oxaliplatin, are associated with a range of adverse effects. These include myelosuppression, oral mucositis, diarrhea, acute cholinergic syndrome, nausea, emesis, neurotoxicity, hand-foot syndrome, cutaneous adverse effects, ocular toxicity, cardiotoxicity, small bowel toxicity, asthenia, elevated liver transaminase levels, and alopecia [19]. The use of specific drugs, alterations in administration schedules, and non-pharmacological methods are recommended for alleviating these adverse [19].

Hesperidin, a flavonoid found in citrus fruits, has been shown to have a significant impact on cancer cells. It interacts with various cellular targets, including CAMKIV, and modulates pathways involved in cell cycle arrest, apoptosis, antiangiogenic, and ant metastatic processes. Hesperidin has been found to inhibit the proliferation of breast cancer cells and androgen-dependent prostate cancer cells, suggesting a potential role in hormone-related cancers. These findings highlight the potential of hesperidin as a natural compound with anticancer properties [6–8].

The size of hesperidin-containing PLGA nanoparticles is crucial for their cellular uptake and therapeutic effectiveness. Smaller nanoparticles tend to have enhanced cellular uptake due to their ability to penetrate biological barriers more effectively. For instance, studies have shown that nanoparticles with a diameter in the range of 100–200 nm are optimal for cellular internalization, leading to increased cytotoxicity against cancer cells [8, 20]. This size range facilitates better distribution within tumors and enhances the therapeutic index by allowing more precise targeting of cancer cells while minimizing effects on healthy tissues.

The composition of the nanoparticles, particularly the ratio of PLGA and the encapsulated hesperidin, also plays a vital role in their performance. Different formulations can affect the release kinetics of hesperidin, which is critical for maintaining effective drug concentrations at the target site over time. For example, variations in the PLGA molecular weight and the presence of additional stabilizers or surfactants can influence the encapsulation efficiency and stability of the nanoparticles [8, 21].

Moreover, the incorporation of other agents alongside hesperidin, such as chemotherapeutic drugs, can lead to synergistic effects that enhance overall cytotoxicity against colorectal cancer cells. For instance, formulations combining hesperidin with 5-fluorouracil have shown improved anticancer activity compared to either agent alone, indicating that the composition can be optimized for better therapeutic outcomes [20, 22].

Due to their high efficacy and lower side effects compared to synthetic drugs, they have shown a remarkable effect in the treatment of malignant and similar diseases. However, the clinical use of hesperidin has been limited due to its low solubility in water and body fluids, low bioavailability and limited absorption. Effective delivery to the target tissue was difficult and limited. Therefore, nanotechnology is used for optimal utilisation and targeted delivery. This technology can overcome the problems of low solubility, bioavailability and absorption of drugs [9–11].

In this article, PLGA nanoparticles were synthesized using the single emulsion evaporation technique, which is a viable method for nanoparticle production [23]. Furthermore, SEM, AFM, FT-IR, DLS, and UV-Vis were employed to ensure the quality of the manufactured nanoparticles.

The MTT assay was used in this study. This assay is used to examine and measure metabolic activity as an indicator of cellular viability, proliferation and cellular toxicity. In this study, it was used to investigate the effects of PLGA nanoparticles containing hesperidin at different concentrations on the survival of HCT116 colorectal cancer cells compared to control groups. The results of the statistical analyses of the MTT assay show that PLGA nanoparticles containing hesperidin were able to reduce the viability of cancer cells. Furthermore, a greater reduction in cancer cell viability was observed at a concentration of 10 µg/ml than at concentrations of 1, 5 and 25 µg/ml nanoparticles.

The size of the PLGA nanoparticles containing hesperidin was approximately 50 nm, with a hydrodynamic diameter of 76.2 nm in the aqueous state. The uniform and spherical nature of the nanoparticles may contribute to their effectiveness in reducing the survival rate of colorectal cancer cells. Additionally, the high drug release rate of around 93% after 144 h indicates efficient delivery of hesperidin to the cancer cells.

Various studies have been conducted on the positive effects of hesperidin in the improvement and treatment of various diseases. In all these studies, the effect of hesperidin on the amelioration of diseases such as cancer has been demonstrated through various mechanisms including induction of apoptosis and cell cycle arrest, inhibition of tumor cell metastasis, angiogenesis, chemoresistance and modulation of oxidative stress. It can be concluded that hesperidin nanoparticles are also able to reduce the percentage of surviving cancer cells, similar to the results of the present study. In 2008, Park et al. conducted a study to investigate the apoptotic effects of hesperidin on human colon cancer cells SNU-C4 at concentrations ranging from 1 to 100 micromoles. Using the MTT assay, they discovered that hesperidin decreased cell viability to 0.05 ± 65.00% of the control values at a concentration of 100 micromolar. The TUNEL assay and DAPI staining were utilized to investigate the apoptotic features brought on by hesperidin [24].

However, in this study, hesperidin was replaced with polymer nanoparticles of PLGA containing hesperidin, which were created and used at 1,5,10 and 25 µg/ml. These nanoparticles had a higher bioavailability than hesperidin. In addition, the MTT assay was utilized to determine the percentage of cancer cell survival, just like in this study, and the HCT116 cell line was used in place of the SNU-C4. The assay results in this investigation showed that, in contrast to the Park study, the concentration of 10 µg/ml hesperidin nanoparticles showed a greater reduction in the percentage of cancer cell survival when compared to other concentrations and control groups, regardless of the dose-dependent effect.

To increase hesperidin’s bioavailability and absorption, a polymer nanoparticle form containing hesperidin was created in the current study as opposed to utilizing hesperidin alone. Furthermore, colon cancer cell line was utilized rather than ovarian cancer cell line. In a study similar to this one, colorectal cancer cells were treated with hesperidin nanoparticles for 48 h as an alternative to 6, 12, and 24 h using the MTT assay to determine the percentage of cancer cell survival.

In contrast to the study by Park et al., the hesperidin nanoparticles in the present study did not show dose-dependent effects on the percentage of cancer cell survival in the MTT assay, and the percentage of colorectal cancer cell survival at a concentration of 10 µg/ml of hesperidin nanoparticles was lower than the concentrations of 1, 5 and 25 µg/ml [24].

In this study, two approaches were pursued: firstly, the development of a suitable Nano delivery system for hesperidin and secondly, its effect on colon cancer cell lines. In various studies, PLGA was used as a Nano delivery for hesperidin, which also showed a good effect. The point of discussion that exists in this study and is a variant to the other studies is that due to the difference of the cell line of this study to the others and also the aggregation of PLGA that occurred which cause the access of cell to PLGA Loaded Hesperidin, the concentration of 10 mg has a better effect. is that this result is different from the result of Park et al.

In 2020, Yang and his colleagues conducted a study in which they synthesized biopolymer nanocomposites based on sodium alginate and pectin containing hesperidin with an approximate size of 200 nm. They investigated the anticancer potential against HCT116 colon cancer cells. They used the MTT assay to measure the effect of the nanocomposites on cancer cells and utilized TUNEL and DAPI staining as well as the reactive oxygen species (ROS) generation method to investigate the anticancer effect of the hesperidin nanocomposites. Flow cytometry analysis confirmed a disruption of mitochondrial membrane potential in the colon cancer cell line. Immunoblot analysis was then performed for caspases 3, 9, PARP, the pro-apoptotic protein Bax and the anti-apoptotic protein Bcl2 [25].

Similar to the study by Yang and colleagues, the MTT assay was also used in the present study to evaluate the effect of hesperidin nanoparticles on the percent survival of HCT116 cancer cells. However, instead of concentrations of 5, 10 and 15 µg/ml, concentrations of 5, 10 and 25 µg/ml were used in this study, which showed no dose-dependent effects of hesperidin nanoparticles in reducing the survival percentage of HCT116 cells, in contrast to the study by Yang et al. The concentration of 10 µg/ml showed a stronger effect in reducing colon cancer cell survival compared to the other two concentrations [25].

In 2021, Balakrishnan et al., synthesized PLGA nanoparticles loaded with hesperidin with a particle size of 4.38 nm. They used the MTT assay to study cellular toxicity and employed DAPI staining, flow cytometry and the HEp-2 cell line to investigate the apoptotic pathway. For the MTT assay, they employed 12 different concentrations, ranging from 8 to 30 micrograms per milliliter, and they treated cancer cells with nanoparticles for a full day (18). Similar to Balakrishnan et al.‘s work, the current study synthesized 50 nm-sized PLGA nanoparticles containing hesperidin. Similarly, the percentage of cancer cells that survived and the cellular toxicity were evaluated using the MTT assay. Nevertheless, the hct116 cell line was utilized in place of the HEp-2 cell line, and hesperidin nanoparticles at varying concentrations—5, 10, and 25 µg/ml—were used. The two primary variations seen in these two research studies are the distinct cell lines targeted and the varied effects produced by the dosage used. Furthermore, hesperidin nanoparticles were applied to cancer cells for 48 h, as opposed to 24 h in the current study [18].

## Conclusion

Our objectives for this work were to create a nanoparticle coating hesperidin that would boost hesperidin’s bioavailability and examine how it affected colorectal cancer cells.

PLGA nanoparticles coated hesperidin appears to solve the problem of its precipitation and lack of solubility in water. It is probable that nanoparticles showed improved efficiency during cell absorption and endocytosis. Hence, the prerequisites for the substance within the nanoparticles to act effectively on cancer cells were improved.

Despite the fact that our research demonstrated the early viability of this Nano particle, further research and testing are required, including real-time testing and flow cytometry-based assays for measuring apoptosis. Following this, the system should also be studied in the animal phase. be positioned. It can be generally concluded that the use of Nano particle as a possible drug delivery system increases the drug’s effectiveness and increases the succeed to win over the illness.

## Data Availability

The raw data required to reproduce these findings are available from the corresponding author upon request.
